# Influence of social media channels on tobacco product consumption behaviors in people aged 18–30 years

**DOI:** 10.3389/fpubh.2026.1718804

**Published:** 2026-02-23

**Authors:** Regina Hanke, Andreas Hoheisel, Kwan-Young Yang, Andrea Kloß, Ivan Cherrez-Ojeda, Daiana Stolz

**Affiliations:** 1 Macromedia University of Applied Science, Berlin, Germany; 2 Clinic of Pneumology, Medical Center-University of Freiburg, Faculty of Medicine, University of Freiburg, Freiburg, Germany; 3 Independent Data Scientist, Bad Homburg vor der Höhe, Germany; 4 Institute for Allergology, Charité – Universitätsmedizin Berlin, Corporate Member of Freie Universität Berlin and Humboldt-Universität zu Berlin, Berlin, Germany; 5 Respiralab Research Group, Guayaquil, Ecuador

**Keywords:** tobacco product consumption, social media usage, vaping, e-cigarette, normative social behavior, social media content, social media channels

## Abstract

**Introduction:**

The use of nicotine through smoking and vaping is a risk factor for chronic diseases. Today, the younger generation (ages 16–25) is potentially exposed to nicotine-related marketing through using social media. Here, we assessed associations between nicotine consumption types and specific social media channel engagement, and whether there is a connection between channels, preferred content, and nicotine consumption type.

**Methods:**

This observational, cross-sectional, online survey was conducted among individuals aged 18–30 via a university’s communication channels in three waves, capturing self-reported social media habits and nicotine use behaviors. Participants confirmed that they were at least 18 years old and proficient in German. The questionnaire was voluntary, pseudonymous, and conducted in accordance with General Data Protection Regulation. Nicotine users and non-users were welcome, and it was assumed that most people had some social media exposure. Only the leading social media platforms were reviewed. Multinomial logistic regressions examined the relationship between smoking status and social media usage intensity, with smokers serving as the dependent variable.

**Results:**

Overall, 300 questionnaires were completed and analyzed. An association was found between nicotine consumption behavior and social media channels, with vapers having the highest use of Instagram (OR, 1.905 vs. non-smokers, OR 1.469 vs. smokers) and X (OR, 1.241 vs. non-smokers, OR 1.409 vs. smokers) compared to other channels. YouTube (OR, 1.774) was the preferred platform for smokers vs. vapers, and Instagram (OR, 1.297) was the preferred platform for smokers vs. non-smokers. Smokers consumed more knowledge and science content (OR, 1.225) and less fashion and beauty content (OR, 0.756) than non-smokers. Smokers consumed significantly more news content (OR, 3.731), and substantially less gaming content (OR, 0.310) and fashion and beauty content (OR, 0.451) than vapers.

**Discussion:**

Our study highlights a potential association between social media use and nicotine consumption in Germany. It raises questions about how the nicotine industry uses visual and linguistic methods to foster addictive behaviors by promoting tobacco accessories, which circumvent the current bans.

## 1 Introduction

Nicotine consumption and vaping are major causes of preventable diseases worldwide, contributing particularly to lung diseases (1). Smoking in any form is a key risk factor for conditions like chronic obstructive pulmonary disease (2). The younger generation (Gen Z, around ages 16–25) is particularly at risk because evidence shows that early nicotine exposure during adolescence or young adulthood is a significant risk factor for later psychiatric disease (3).

A study by the DEBRA Study Research Group, focusing on adolescents and young adults, has shown that tobacco product consumption in Germany has increased in the last years. The results showed an increase in the number of consumers, both among smokers and increasingly among e-cigarette users (hereafter referred to as vapers) (4). This is contrary to trends seen in previous years, but confirms the increasing use of vaping observed (5). These results are supported by the sales of vapes, which rose from €330 million in 2020 to €800 million in 2023, and the slight decline in cigarette sales, from €22.8 million to €21.8 million, during the same period (6).

In recent years, the global use of social media has increased significantly, particularly among young people. Overall, usage averages 1.4 h per day, with Gen Z spending 2.5 h per day on these platforms. The leading networks include YouTube, Facebook, WhatsApp, Messenger, TikTok, and Instagram. While Facebook is the second-largest social media network, it also has the oldest user group, with over 17% of users falling within the 55–64 age range (7).

Concurrently, exposure to divers forms of nicotine marketing has also evolved. Although Germany enacted bans on nicotine advertising, such as outdoor billboards, many young people still report encountering nicotine or vaping promotions. Some leading social media platforms have committed themselves to banning the advertisement of cigarettes, vapes or any tobacco and nicotine products from their platforms. Still, the current legislation leaves the option open for influencers, festivals, or a ‘brand stretch,’ where non-nicotine-related products (such as paper, vape accessories, and fashion accessories) can be marketed. A 2020 survey by the European Commission showed that 49% of the German population reported seeing advertisements for cigarettes, and 42% encountered advertisements for vapes or liquids within the last 12 months (8).

A more recent survey by Cavazos-Rehg et al., conducted in the United States, highlights the positive correlation between active and passive interaction with social media marketing campaigns for nicotine-related products (9). Social media consumption increased the likelihood of initiating the use of nicotine products.

Given the rapid increase in social media engagement and the nicotine industry’s subtle online marketing tactics, it is essential to understand where and how young people might be influenced toward nicotine consumption. This knowledge can inform effective health communication campaigns that counter pro-nicotine social influence in the digital arena, potentially leading to more effective prevention or cessation programs.

The current study aimed to assess whether there is an association between the type of nicotine consumption (non-smoking, smoking or vaping) and the nature of specific social media channel engagement among young adults (aged 18–30). We also evaluate whether there is any connection between the channel, preferred content and the type of nicotine consumption.

## 2 Methods

### 2.1 Study design and setting

This was an observational, cross-sectional, online survey of individuals in the target age range of 18–30 years. The observational design aimed to capture a snapshot of social media habits and nicotine use behaviors in the population at a single point in time. Data was collected via a web-based questionnaire using the LimeSurvey platform. The survey was distributed via the university’s communication channels, such as the cafeteria and word of mouth, as well as through non-university activities.

Study recruitment took place in a university setting and public venues in Berlin, Germany, in three waves. It was voluntary and pseudonymous; no personal data were collected in Waves 1 and 2. In Wave 3, participants could provide their matriculation number and name voluntarily.

The three survey waves took place from mid-2023 to mid-2024 to maximize their reach. Each wave corresponded to an academic semester, leveraging student involvement and outreach during that period. This allowed for iterative revisions following an initial pilot to improve clarity and structure and ensured sufficient sample sizes throughout the study period.

In Wave 1, to reach beyond the university, flyers and posters were placed in public premises frequented by young people. Wave 2 continued with a similar outreach on a smaller scale. To boost participation, in Wave 3, university students were offered a small incentive: a course credit certificate worth 0.2 points for completing the survey. Participants could voluntarily provide their name and student ID at the end of the questionnaire to receive credit; any identifying information was stored separately and later removed from the research dataset to maintain anonymity.

### 2.2 Study population

The study recruited adolescents and young adults aged 18–30. Participants had to confirm they were at least 18 years old before beginning the survey (the survey welcome text stated you can participate “if you are older than 17 years,” i.e. 18+). Participants needed sufficient proficiency in the German language to comprehend the questions.

Individuals under 18 were excluded through the consent requirement. There was no upper age cutoff programmed, but recruitment materials targeted individuals under 30. Participation was not restricted by gender, education, or smoking status. Both nicotine users and non-users were welcome, and recruitment primarily targeted students and young professionals in urban Germany. It was assumed that most people in this demographic had some exposure to social media.

### 2.3 Questionnaire content

The final version of the questionnaire is included in Appendix 1. The questionnaire consisted of multiple sections that captured the following key information: demographics (age, gender and education); nicotine use behavior (current non-smoker, smoker or vaper and frequency of use: daily, occasionally, not at all); social media usage and frequency using a 5-point Likert scale (1 = never, 5 = very frequent/daily) and the following platforms: Instagram, TikTok, YouTube, Twitter (X) and Facebook; social media content engagement (how often do you engage with content related to news, gaming, entertainment, fashion and beauty, and knowledge/educational content? With the following ratings on a 4-point Likert scale: 1 = not at all, 4 = a lot); additional behavioral questions (average time spent on social media [<1 h, 1–2 h, 2–4 h, 4 + hours], whether participants recall seeing nicotine-related content on social media (yes/no), and open-ended questions for any comments on how social media might influence smoking/vaping habits).

Only the leading social media platforms with high image and video content were reviewed, where users receive a highly personalised feed sampled by an algorithmic approach. WhatsApp and other messengers were excluded due to the lack of common social media features, automated personalised feeds and deep integration of images and video content.

### 2.4 Statistical analysis

**Figure 1:**
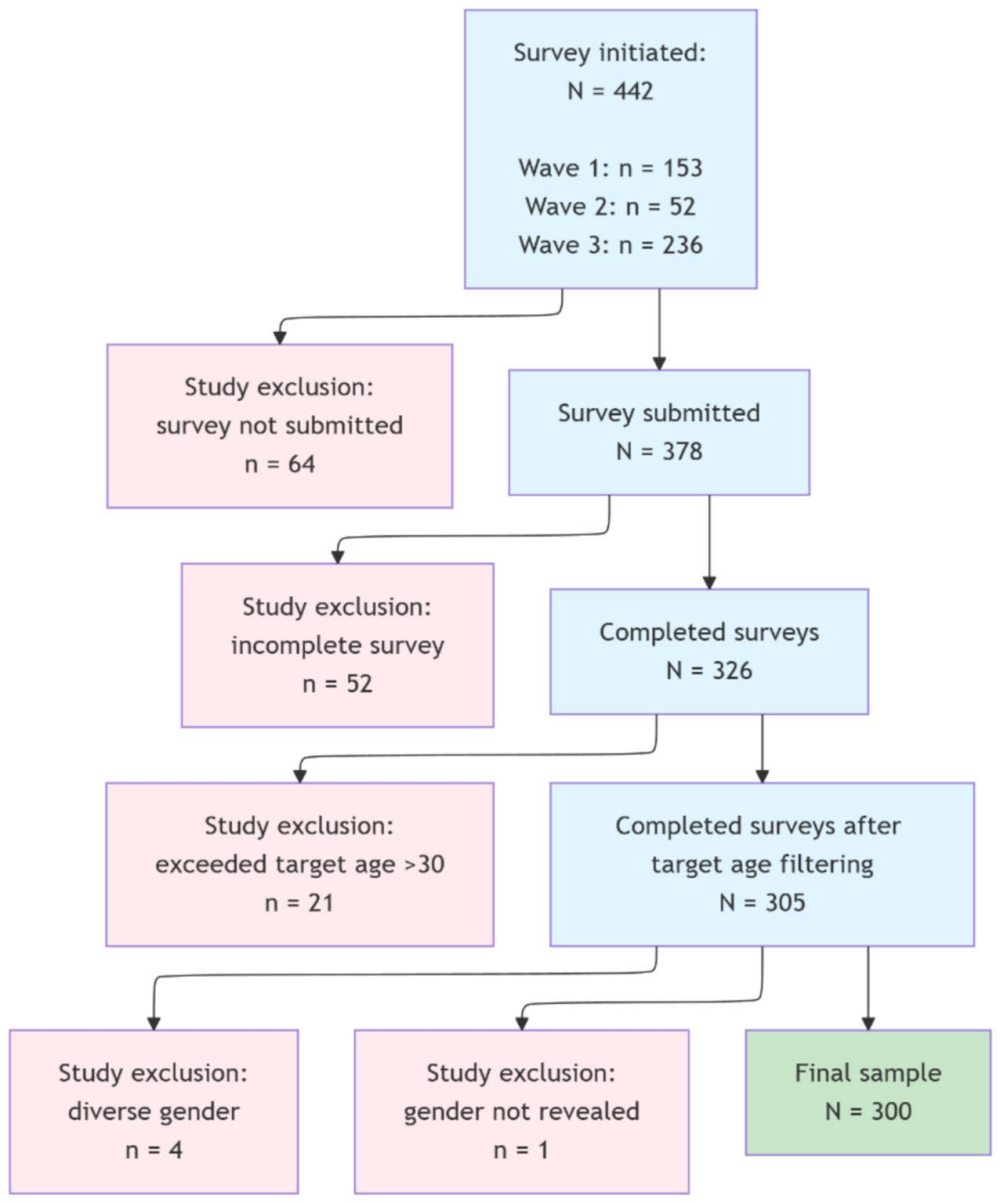
Flow chart sample composition.

Responses that were incomplete due to answering questions only regarding demographics but not the substantive sections of social media or smoking behavior were excluded from the final analysis. Table 1 provides an overview of questions with incomplete answers that were included in the final sample. Out of 378 submitted surveys, 52 did not answer any questions regarding their social media usage habits. 21 respondents exceeded the target age of >30, and 5 indicated gender that were unambiguously classifiable, with 300 qualifying for the final sample. The final sample contains one response that answered “Hookah” for “other” for their smoking status group categorization. This case also identified as a smoker and has been categorized as such. The exact breakdown of the sample and exclusion criteria is visualized in Figure 1. Any duplicate entries (e.g., the same person taking the survey twice) were excluded. However, the anonymous design and distribution method made duplicate detection possible only through self-reported or obviously identical demographic combinations, which did not occur.

**Table 1:** Inclusion of questions with incomplete answers.

	Queries	Responses	Missing	Missing %
Level of education	300	204	96	32.0%
Hazard level estimate in general	300	234	66	22.0%
Hazard level estimate for self	300	299	1	0.3%
Financing	105	103	2	1.9%
Estimate of the number of chemicals	300	268	32	10.7%
Use of Google	300	299	1	0.3%

For the primary analyses, we conducted multinomial logistic regressions to examine the relationship between smoking status group membership (non-smokers, smokers, and vapers) and the intensity of social media usage. Smoking status group membership served as the dependent variable. We estimate the odds ratios (OR) with 95% confidence intervals (CI) for each predictor variable. To facilitate interpretation of all pairwise comparisons, we ran separate models with non-smokers and vapers as reference categories.

The following were used as predictor variables: 1. Social media platforms (consumption level measured between 1 and 5): TikTok, YouTube, Twitter, Instagram, Facebook; 2. Content types (consumption levels measured between 1 and 4): Knowledge, Gaming, News, Entertainment, Fashion & Beauty, and 3. Control variables: Gender (Male as baseline, Female; Supplementary Table 1).

An exploratory analysis was conducted using cross-tabulations to examine the distribution of the responses within the dataset. For Likert-scale variables, we performed one-way ANOVAs (Table 2) to compare the means across the three smoking status groups at a 5% significance level. We assessed normality assumptions through Q-Q plots of standardized and unstandardized residuals from univariate General Linear Models, with smoking status as the grouping factor and each social media platform usage and content type consumption variable as the dependent variable. Twitter and Facebook violated normality assumptions, and Kruskal-Wallis H tests were performed as a non-parametric alternative for these variables. Since the analyses with Twitter and Facebook variables were insignificant, no follow-up was conducted. Homogeneity of variances was assessed using Levene’s Test, and post-hoc pairwise comparisons were conducted using Tukey HSD when variances were equal and Games-Howell when variances were unequal. Additionally, a chi-square test of independence was performed to assess whether the gender distribution differed across smoking status (χ^2^ = 2.60, df = 2, *p*-value = 0.272), which showed no significant association (Supplementary Tables 2, 3).

**Table 2:** Descriptive statistics for platform use and content consumption by smoking status.

		Sum of squares	df	Mean square	F	*p*-value
Tik Tok	Between groups	8.105	2	4.053	2.503	0.084
	Within groups	480.812	297	1.619		
	Total	488.917	299			
YouTube	Between groups	3.496	2	1.748	1.697	0.185
	Within groups	305.901	297	1.030		
	Total	309.397	299			
Twitter^a^	Between groups	0.015	2	0.007	0.035	0.965
	Within groups	62.305	297	0.210		
	Total	62.320	299			
Instagram	Between groups	4.387	2	2.193	2.948	0.054
	Within groups	220.983	297	0.744		
	Total	225.370	299			
Facebook^a^	Between groups	0.019	2	0.010	0.095	0.909
	Within groups	29.728	297	0.100		
	Total	29.747	299			
Knowledge	Between groups	1.044	2	0.522	1.095	0.336
	Within groups	141.543	297	0.477		
	Total	142.587	299			
Gaming	Between groups	26.921	2	13.460	12.924	<0.000*
	Within groups	309.329	297	1.042		
	Total	336.250	299			
News	Between groups	30.500	2	15.250	21.947	<0.000*
	Within groups	206.380	297	0.695		
	Total	236.880	299			
Entertainment	Between groups	0.567	2	0.283	0.649	0.523
	Within groups	129.683	297	0.437		
	Total	130.250	299			
Fashion & Beauty	Between groups	8.005	2	4.003	3.661	0.027*
	Within groups	324.742	297	1.093		
	Total	332.747	299			

a For Twitter and Facebook, normality assumption violated.

### 2.5 Data preparation and software

Data was retrieved in CSV format from Lime Survey. Data preparation and initial analysis were conducted using Microsoft 365’s Excel. Data preparation encompassed removing incomplete or missing data, removing all provided student matriculation numbers and names of Wave 3 for pseudonymization, merging the datasets from multiple questionnaire waves, consolidating disjointed questions in similar question categories across groups into a single variable, transforming nominally closed question responses from nominal string responses into Likert-scale or Likert-scale-like data, and unifying response scales into comparable magnitudes. Open-ended question responses were coded and categorized. The data were then transferred and coded into IBM’s SPSS Statistics (version 25) for multinomial logistic regression analysis.

Other statistical tests were performed within Excel using custom VBA programs. Data visualization was conducted using Excel and Python, with the aid of the pandas, NumPy, and matplotlib libraries. For higher resolution and visual quality, the data was generated in Adobe Illustrator.

### 2.6 Ethics statement

The studies involving human participants were reviewed and approved by the Ethics Committee of Macromedia University of Applied Sciences (reference number: 2025-2). All participants provided written informed consent to participate in this study before participation. The study was conducted in accordance with the Declaration of Helsinki, the Good Clinical Practice Guidelines, German law, the regulations of the German regulatory authorities, and in compliance with the General Data Protection Regulation.

## 3 Results

### 3.1 Study population demographics

**Figure 2:**
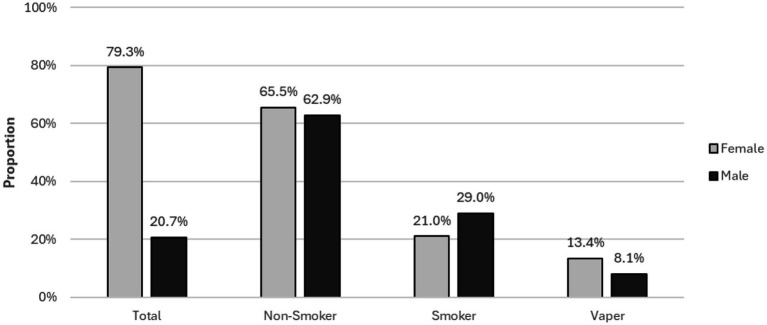
Demographics and gender.

In total, 442 participants took part in the study, with 300 completing the questionnaire fully. The survey was conducted in three waves: Wave 1, summer semester 2023 (*n* = 96), Wave 2, winter semester 2023/2024 (*n* = 25), and Wave 3, summer semester 2024 (*n* = 179). The demographic distribution of the participants (*N* = 300) is reported in Table 3. The gender structure is presented in Figure 2. Only complete data sets are included in the analysis.

**Table 3:** Baseline demographic characteristics and education level of the study population.

	*n* (%) *N* = 300
Gender	300
Male	62 (20.7%)
Female	238 (79.3%)
Educational level	204 (68.0%)
Lower secondary school	1 (0.5%)
Intermediate secondary school	1 (0.5%)
Upper secondary school (no university entrance qualification)	1 (0.5%)
University entrance qualification	15 (7.4%)
University student	179 (87.7%)
Completed university degree	5 (2.5%)
Non-academic occupation	2 (1.0%)
No response	96 (47.1%)
Non-Smoker (no consumption of cigarettes or vapes)	129 (43.0%)
Lower secondary school	1 (0.8%)
Intermediate secondary school	1 (0.8%)
Upper secondary school (no university entrance qualification)	0 (0.0%)
University entrance qualification	9 (7.0%)
University student	112 (86.8%)
Completed university degree	4 (3.1%)
Non-academic occupation	2 (1.6%)
No response	66 (51.2%)
Smoker (consumption of Cigarettes)	45 (15.0%)
Lower secondary school	0 (0.0%)
Intermediate secondary school	0 (0.0%)
Upper secondary school (no university entrance qualification)	0 (0.0%)
University entrance qualification	1 (2.2%)
University student	43 (95.6%)
Completed university degree	1 (2.2%)
Non-academic occupation	0 (0.0%)
No response	23 (51.1%)
Vaper (consumption of vapes)	30 (10.0%)
Lower secondary school	0 (0.0%)
Intermediate secondary school	0 (0.0%)
Upper secondary school (no university entrance qualification)	1 (3.3%)
University entrance qualification	5 (16.7%)
Vocational training/apprenticeship	0 (0.0%)
University student	24 (80.0%)
Completed university degree	0 (0.0%)
Non-academic occupation	0 (0.0%)
No response	7 (23.3%)

### 3.2 An association exists between nicotine consumption behavior and social media channels

**Figure 3:**
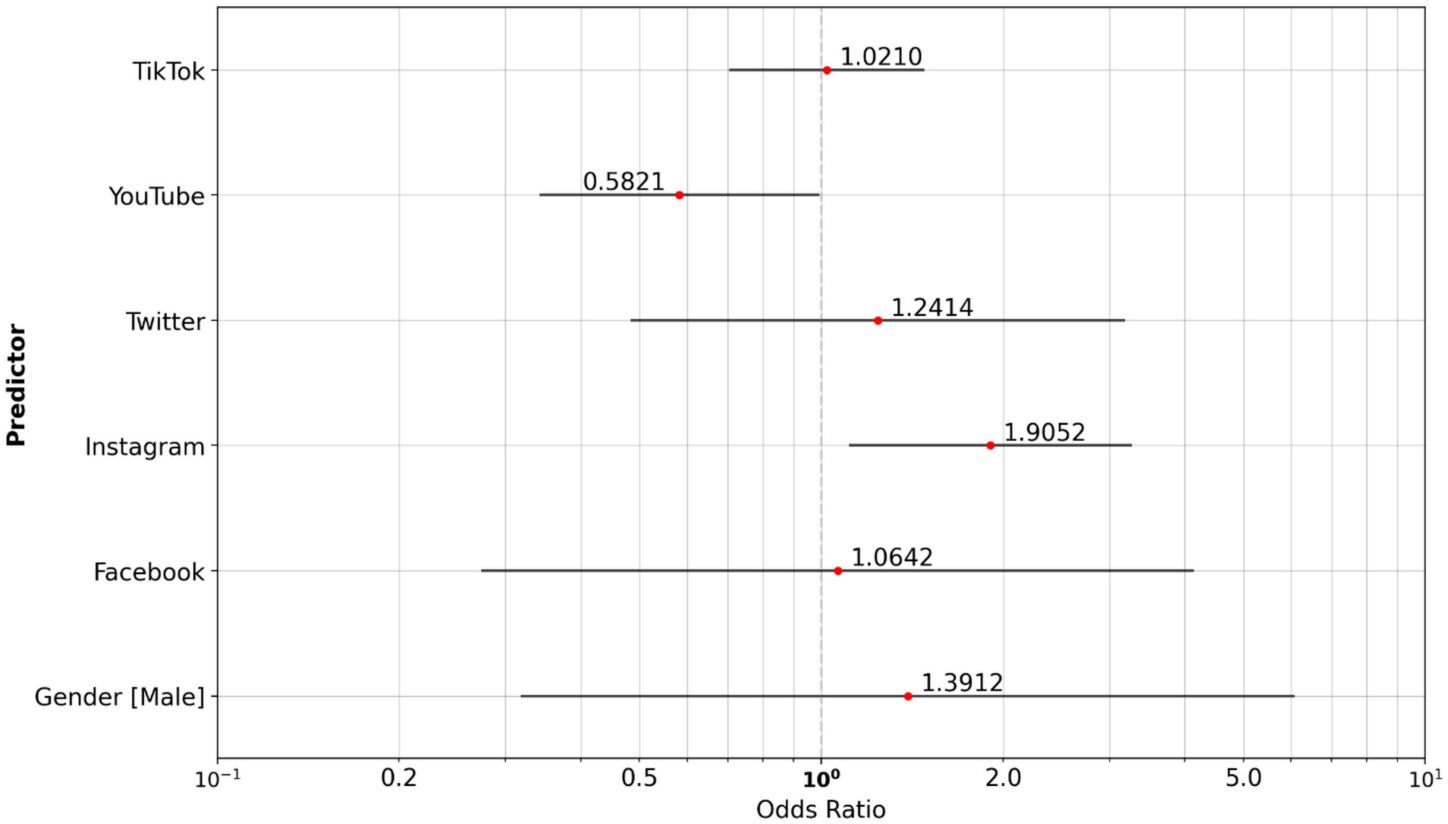
Odds ratio social media: non-smoker reference group vs. vaper group.

A significant association exists between the vaper group and the non-smoker reference group. The study population that vapes has the highest usage of Instagram (OR, 1.905) and a slightly increased use of X (OR, 1.241; Supplementary Table 3) compared to other social media channels (Figure 3).

In the multinomial logistic regression analyses (Supplementary Table 3) using non-smokers as the reference group, vapers showed statistically significant associations with several platform-use and content-consumption variables. Specifically, vapers were associated with Instagram use (OR = 1.905, 95% CI 1.112–3.264, *p* = 0.019), YouTube use (OR = 0.582, 95% CI 0.341–0.992, *p* = 0.047), gaming-related content consumption (OR = 2.756, 95% CI 1.703–4.462, *p* < 0.0001), and news-related content consumption (OR = 0.284, 95% CI 0.159–0.505, *p* < 0.0001). In contrast, no statistically significant associations were observed for smokers relative to non-smokers across platform-use or content-consumption variables (all *p* > 0.05). In direct comparisons between smokers and vapers, statistically significant associations were observed for gaming-related content (OR = 0.310, 95% CI 0.179–0.538, *p* < 0.0001), news-related content (OR = 3.731, 95% CI 1.963–7.092, *p* < 0.0001), and fashion and beauty content (OR = 0.45, 95% CI 0.24–0.87, *p* = 0.0166). All other smoker–vaper contrasts were not statistically significant. Gender was not statistically associated with smoking status in any comparison (all *p* > 0.409).

### 3.3 YouTube is the preferred social media platform amongst cigarette smokers

**Figure 4:**
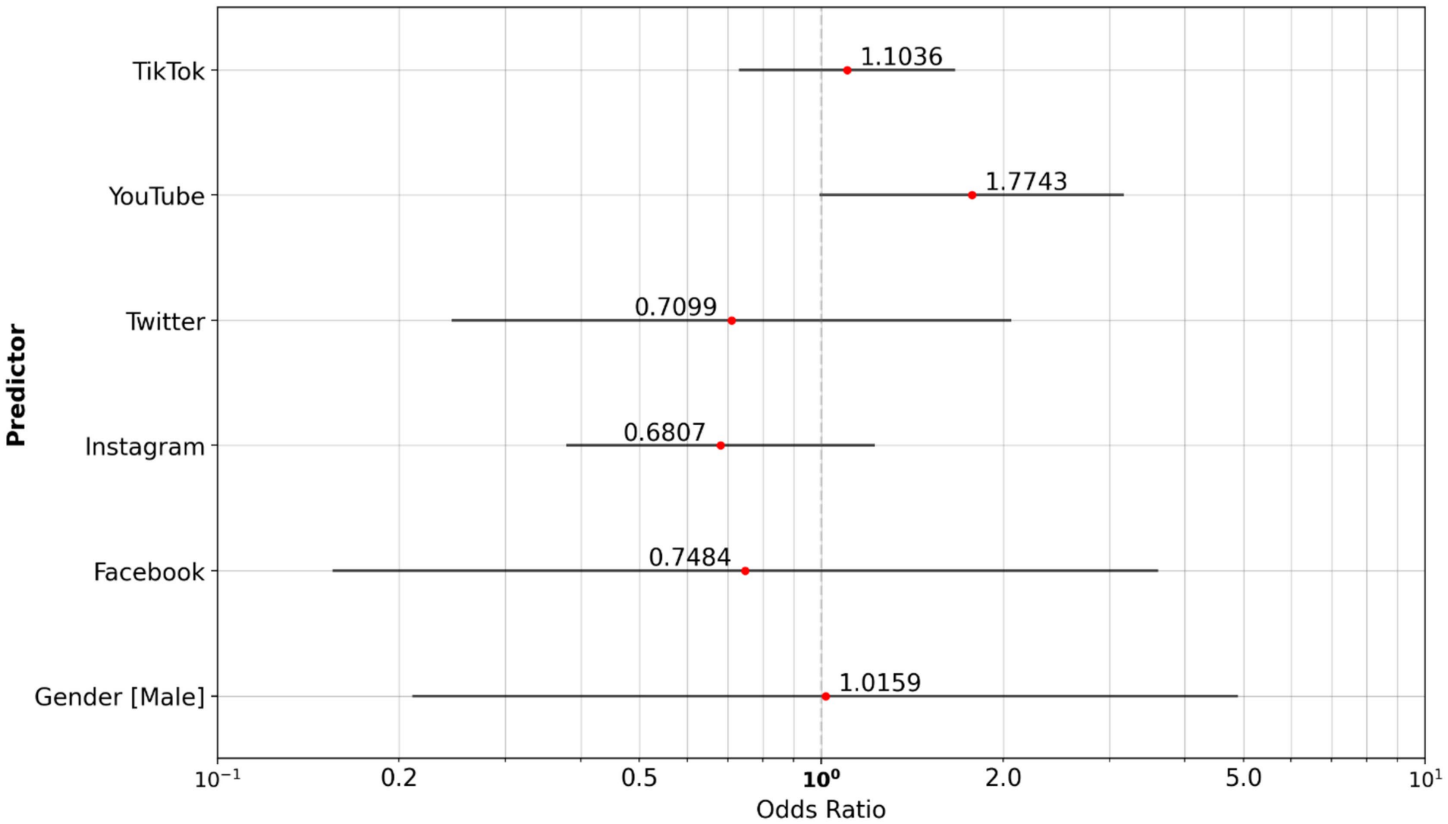
Odds ratio social media: vaper reference group vs. cigarette smoker group.

We compared social media usage between cigarette smokers and vapers. Analysis highlights that cigarette smokers in the study population are more likely to use YouTube (OR, 1.774) as their preferred social media channel (Figure 4).

### 3.4 Instagram is more likely to be used by cigarette smokers than non-smokers

**Figure 5:**
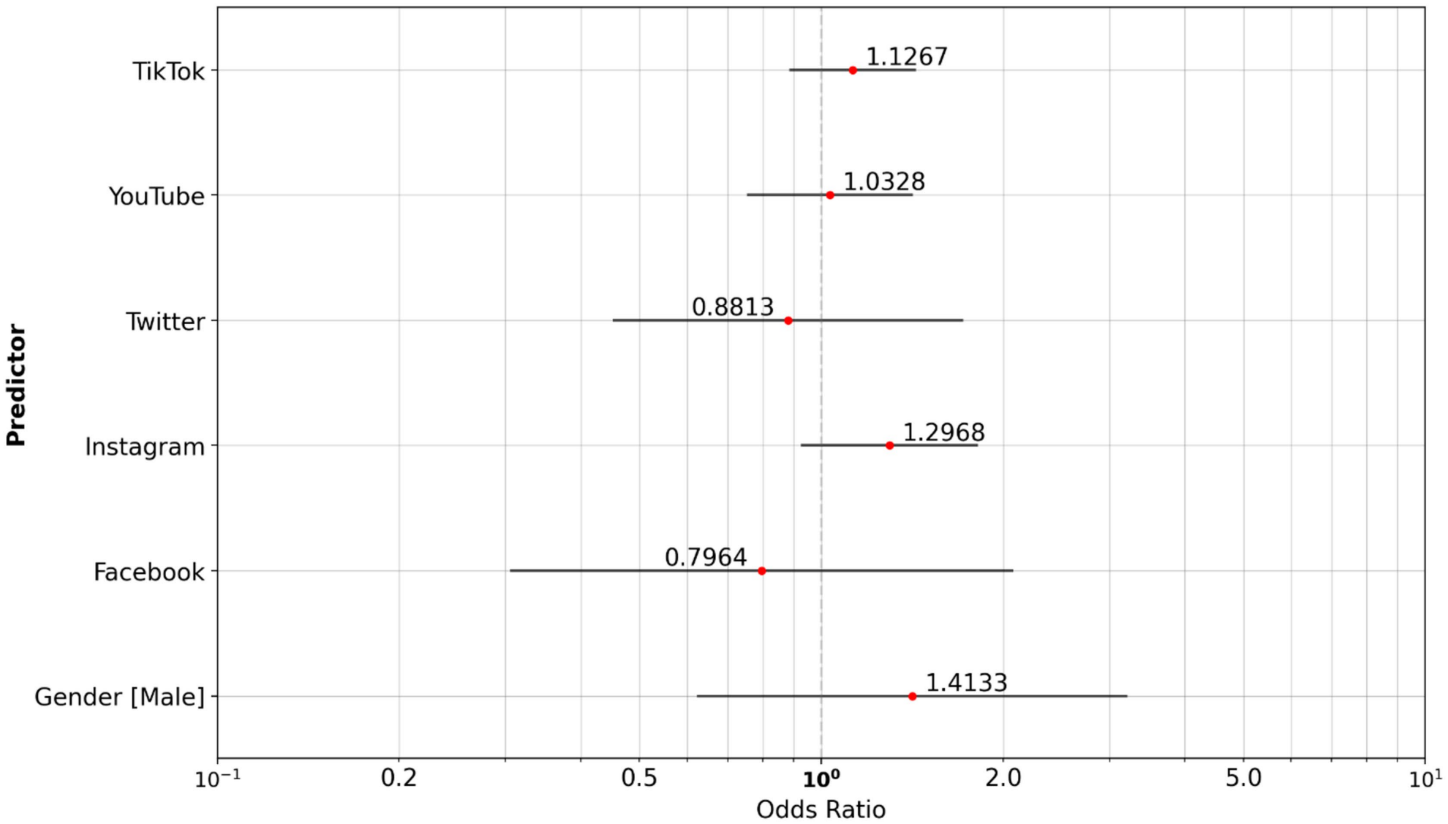
Odds ratio social media: non-smoker reference group vs. cigarette smoker group.

There was a modest association between certain social media platforms (Instagram OR, 1.297) amongst cigarette smokers compared to the non-smoker reference group (Figure 5).

### 3.5 Social media content consumption differs between cigarette smokers and non-smokers

**Figure 6:**
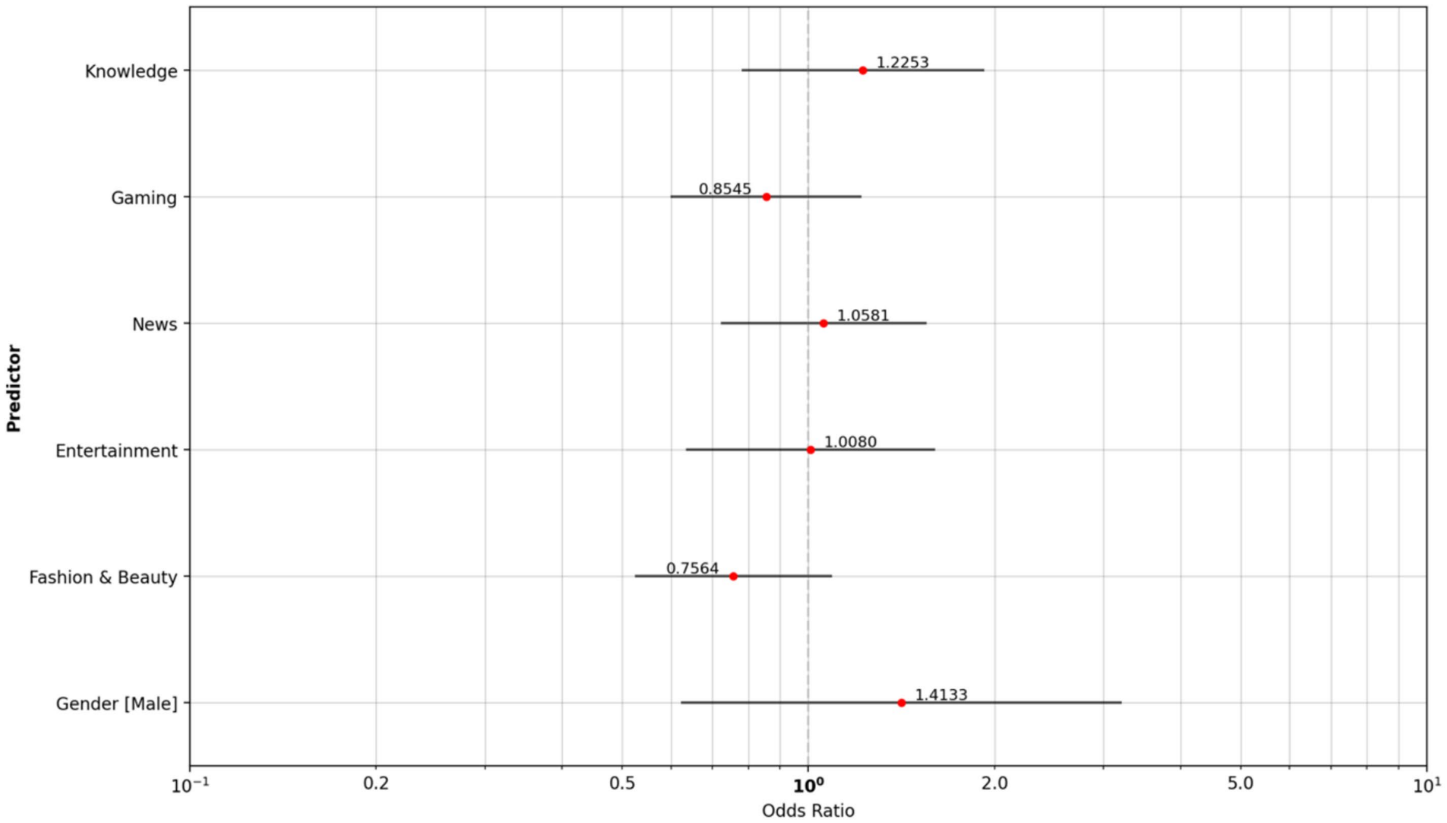
Odds ratio content type: non-smoker reference group vs. cigarette smoker group.

The smoker’s group consumed a little more knowledge and science content (OR, 1.225) and less content around fashion and beauty (OR, 0.756) than the non-smoker reference group (Figure 6).

Supplementary Table 1 shows basic summary information for each group, including how many participants were in each group and the average level of use for each platform or content type, along with measures of variability. The descriptive analysis shown in Supplementary Table 1 confirms this higher consumption, with a mean of 2.985 for smokers who consumed knowledge content, compared with non-smokers (2.882) and vapers (2.284).

Supplementary Table 2 highlights limited but statistically detectable differences in media use across smoking groups. Vapers show distinct patterns in select social media channels compared with Smokers and Non-Smokers.

### 3.6 Social media content consumption differs between cigarette smokers and vapers

**Figure 7:**
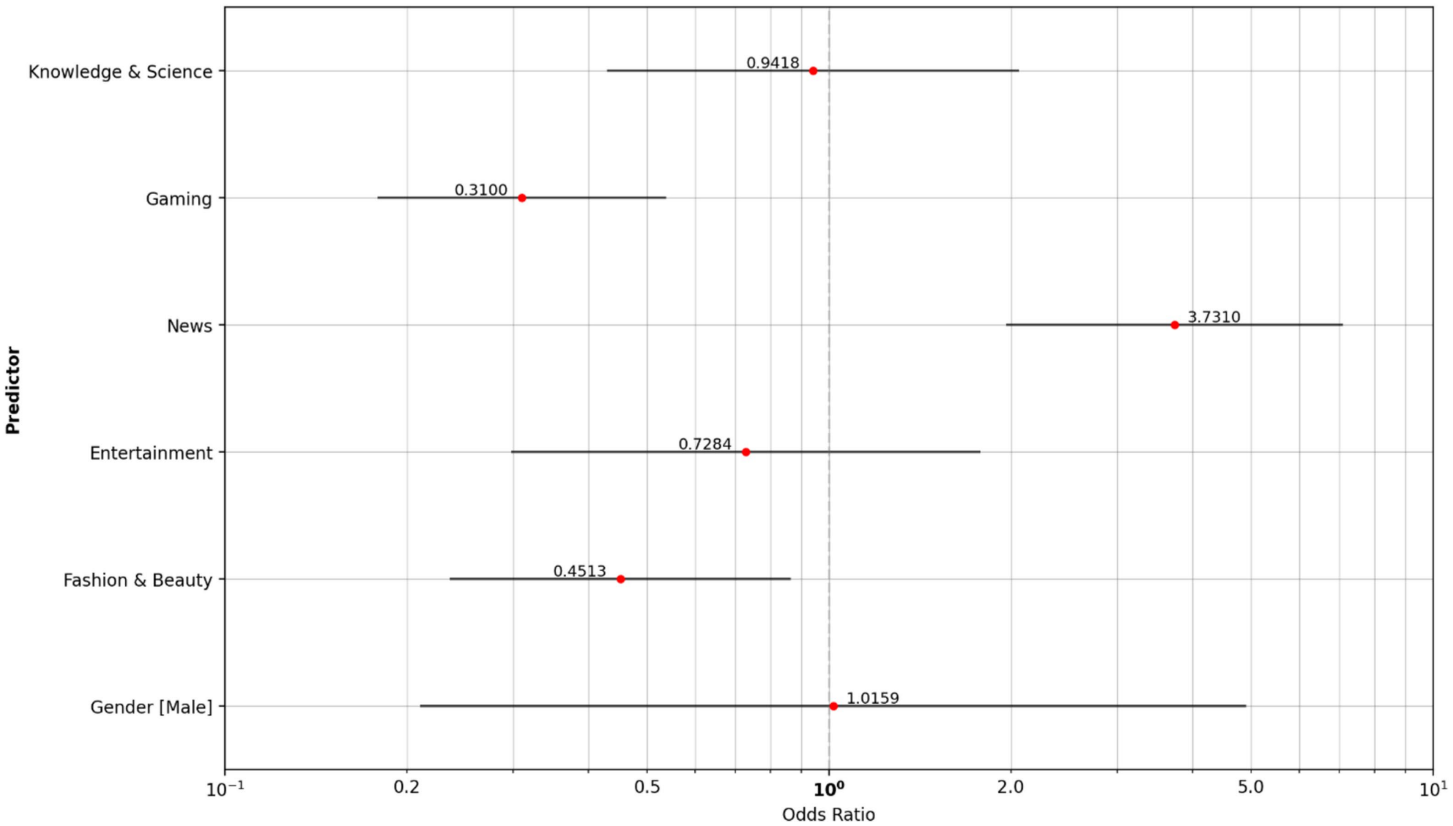
Odds ratio content type: vaper reference group vs. cigarette smoker group.

The smoker’s group consumed significantly more news content (OR, 3.731), and substantially less gaming content (OR, 0.310) and fashion and beauty content (OR, 0.451) than the vapers (Figure 7).

### 3.7 Social media content consumption differs between reference group non-smokers and vapers

**Figure 8:**
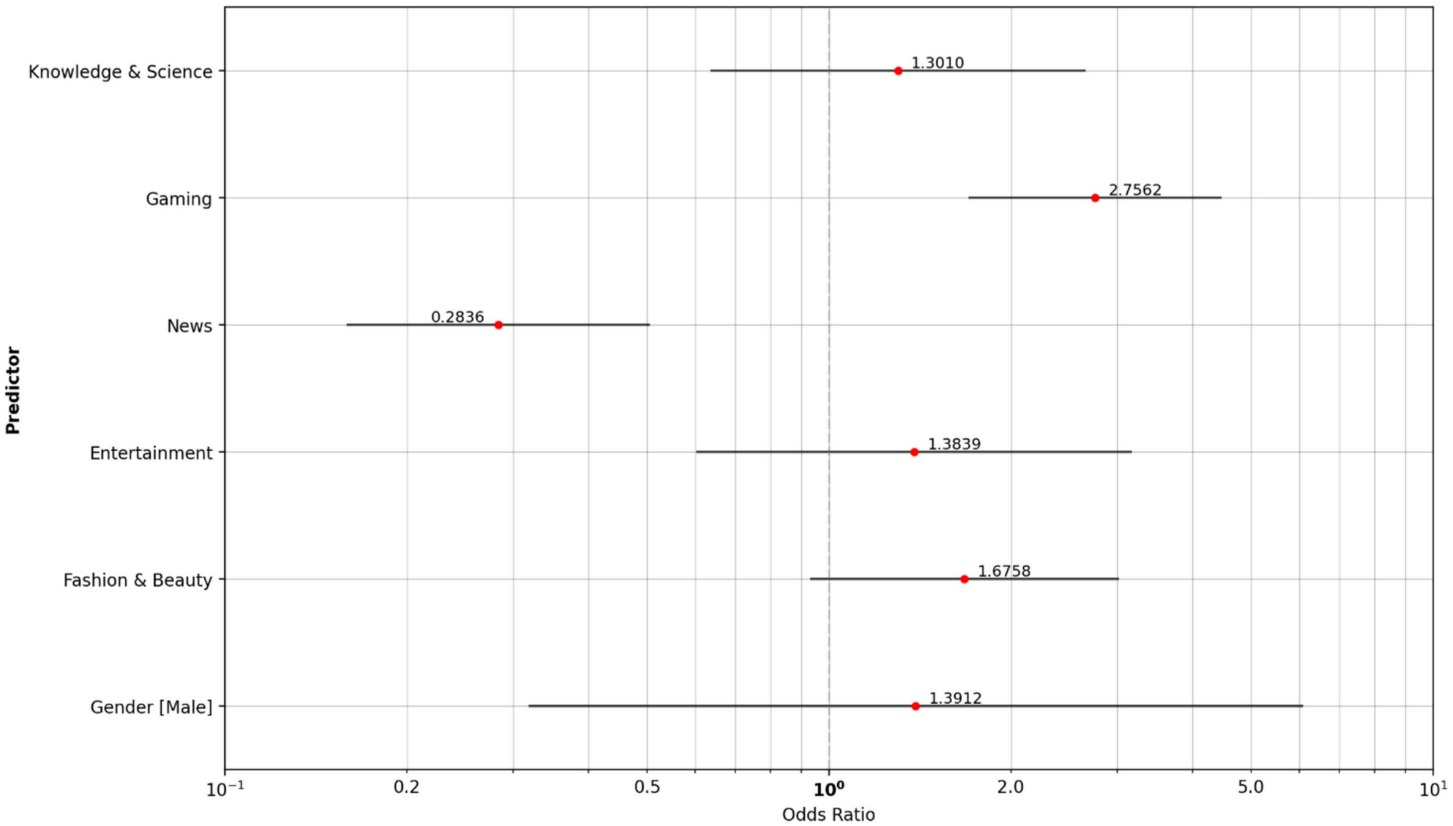
Odds ratio content type: non-smoker reference group vs. vaper group.

The vaper group consumes significantly more gaming content (OR 2.756) than non-smokers, whereas news content has lower odds (OR 0.284) in the vaper group (Figure 8).

## 4 Discussion

A survey from Cavazos-Rehg et al. (9) conducted in the USA, where social media advertisement and campaign activities for electronic nicotine delivery systems (ENDS) and nicotine-related products are allowed, highlights the positive correlation between active and passive interaction with social media nicotine-related product marketing campaigns. Social media consumption and tobacco increased the likelihood of initiating the use of tobacco and or nicotine products [Cavazos-Rehg et al. (10), p. 493]. A scoping review in anglophone countries reports a potential positive relation between social media consumption and ENDS products (10). Our study is supported by research which also demonstrates a correlation between social media consumption and all kinds of nicotine products and non-nicotine end products (9, 11–13). Yet none of those studies looked into a possible correlation or association between social media channel, content consumption and self-reported smoking and/or vaping, e-cigarettes. Additionally, those studies were conducted in countries where marketing activities around Nicotine and ENDS products are not banned.

Despite the ban of ENDS marketing and campaigns and an advertising ban on social media for all kinds of nicotine products and non-nicotine end products in Germany, the results of this study highlight a potential association between specific social media channels and the type of nicotine product consumption in Germany. The results hint that Gen Z vapers are using Instagram significantly more as their preferred social media channel. Gen Z smokers are more likely to be using YouTube, with a slightly higher frequency and duration than the vapers and non-smokers. Gen Z non-smokers have a lower overall social media consumption than vapers and smokers. Of course, this is not a causative link between the use of nicotine and social media, but rather a correlation between the two.

With the recent decline in people smoking and adopting vaping as an alternative, scientific and policy debate continues about how vaping exposure can be controlled due to the unknown long-term health effects of these products. Evidence from previous studies demonstrates that social networking sites can influence nicotine behaviors (10). Germany has banned such promotions, but loopholes remain, and such advertising is still allowed in other countries. Some marketing strategies include sponsorship of celebrities and social media influencers, which can be seen as fostering “normative social behavior”, such as the use of vapes amongst young people. This indicates an urgent need for tighter marketing regulations and revision of communication strategies and touchpoints for ‘Cessation campaigns’.

One study showed that school education, peer group and family role models are other factors for nicotine consumption (12). This study underscores the need for targeted, evidence-based health communication strategies and policy interventions to mitigate nicotine promotion on social platforms. These findings suggest that the nicotine industry effectively addresses target audiences to create nicotine consumer groups. Even though there are already advertising bans in place in Germany, the content on platforms like Instagram and TikTok is correlated with vaping. In contrast, YouTube content correlates more with cigarette smoking. We must understand that visual and linguistic as well as brand-stretch methods are used to exert influence over people who consume this content. Further research into the visual and linguistic methods used by the nicotine and vaping industry should be conducted to develop appropriate countermeasures to reduce preventable lung diseases.

Our study presents interesting new evidence that certain social media sites, particularly Instagram, are more prevalent among young people, corroborating previous evidence that suggests social networking sites may be linked to positive attitudes towards vaping through user engagement with promotional content (10). Other research by Frey and Friemel points to the normative impact of Snapchat and Instagram on tobacco use, suggesting that these social media channels increase the chances of vaping or smoking (11). Exposure to this type of content undoubtedly impacts how young people behave and may even encourage them to experiment with vaping or smoking.

We demonstrate that individual participants who vape consume less news content on social media platforms such as Instagram, TikTok, and YouTube compared to smokers and non-smokers. Conversely, vapers in the study population exhibit a higher consumption of gaming-related content. Additionally, the study reveals that a significantly larger proportion of vapers engage with beauty content compared to smokers and non-smokers, which may be attributed to the higher ratio of women to men among vapers within this survey. The type of social media platform and the content being consumed play a significant role in influencing the probability of vaping or smoking. There is a clear link between the specific platform, content, and the likelihood of engaging in vaping or smoking. This information is important because it enables public health anti-nicotine campaigns to be directed through the most appropriate channels to target specific user populations, such as female vapers, who can be effectively reached through Instagram and beauty-related content.

The results of this study suggest that information or cessation campaigns in the context of nicotine use should consider the following aspects: (1) Information and cessation campaigns in the context of vaping should primarily be posted on Instagram in the contextual environment of beauty and fashion, as well as gaming. Additionally, aspects of content-related messages and visual language should be included. (2) Information and cessation campaigns focusing on smoking should primarily be integrated on YouTube with a contextual focus on news and gaming. However, special attention must be paid to potential reactions and unintended effects to minimize the risk of campaigns for non-nicotine consumers.

### 4.1 Study limitations

Our study has several limitations, primarily related to the low numbers of smokers (22.7%) and vapers (12.3%) included. Females are overrepresented at 79.3% of the study participants. Most participants (65.0%) were non-smokers. The distribution at a university and in university-associated peer groups means that students or people with higher education are more likely to be represented. Therefore, a future study should also include people with lower incomes and a higher proportion of people with non-academic qualifications. In addition, only certain social media channels were available in the selection. The nature of an online survey means that we are reliant on participants to provide accurate information, and demographic data based on voluntarily provided pseudonymous information cannot be verified. Furthermore, a comparison group comprising individuals over 30 should be considered.

## 5 Conclusion

This is the first study to analyze the individual, channel and content-related social media activities of the 18–30 age group, indicating the placement of potential prevention campaigns. Based on these study findings, the question arises how the nicotine industry uses visual and linguistic methods to foster and lay the ground for addictive behaviors, and the role of brand stretch by providing accessories in the context of tobacco product consumption, which circumvent the current bans.

## Data Availability

The datasets presented in this article are not readily available because the study is based on an anonymous questionnaire. Requests to access the datasets should be directed to r.hanke@macromedia.de.
