# Supplementary material for: Influence of social media channels on tobacco product consumption behaviors in people aged 18–30 years
Source: Front Public Health. 2026 Feb 23;14:1718804. doi: 10.3389/fpubh.2026.1718804 (PMC12968281; doi:10.3389/fpubh.2026.1718804)
Supplement: Supplementary file 2 [file Table_2.docx]

**Appendix 2: Informed Consent**

**Human Participants:**
In the realm of the Welcome Text of the Survey and in the Privacy Policy Text the potential publication was stated and hence has been part of the consent box.

**WAVE 1 & 2 Summer Semester 2023 & Winter Semester 2023/24**
**Introduction Text:**
Hello! It's nice that you're supporting us by taking part in our survey! We are conducting an anonymous study on smoking and vaping. You can help us by completing this short questionnaire. There is no ‘right’ or ‘wrong’ answer – just answer the questions as best as you can based on your opinion and knowledge. You will, of course, remain anonymous. You can take part if you are over 17 years old! Very important: The survey is anonymous – that means we don't know who participated. We don't ask for your name, your place of residence, your email address or any other personal information. But we do ask about age, social media use and nicotine consumption – i.e. smoking or vaping. **The results are intended for scientific publications to clarify fundamental questions of communication**. So you are doing something for science! The following scientists are asking: Prof. Regina Hanke (Design Professor at Macromedia - University of Applied Science Berlin), Dr. Andreas Hoheisel (Specialist in Pneumology, Pneumological Practice Leipzig / University Hospital Freiburg (Brsg.), Kwan-Young Yang (Statistician, Bad Homburg vor der Höhe) PD Dr. Thomas Köhnlein (Specialist in Pneumology, Pneumological Practice Teuchern). Thank you for completing the questionnaire – it should take about 10 minutes. Prof. Regina Hanke

**Privacy Policy Text:**
This is a survey as part of a scientific study. The questionnaire will be answered anonymously. We do not collect any data that would allow us to identify you – no email, no names, no addresses – you answer anonymously. Duration of storage: In accordance with good scientific practice, the collected data will be stored anonymously for up to 10 years on the servers of Macromedia – University of Applied Science. The server is located in Germany. Use of the collected data: All anonymously collected data will be used for the following purposes within the framework of the scientific study: Project-related work, Scientific publications, Publications or exhibitions.

**Legal basis:** DS-GVO Art. 13 By participating in the questionnaire, you consent to the processing of your anonymous data. Responsible: Prof. Regina Hanke Design & Design management
r.hanke@macromedia.de

**Privacy Policy Error Text:**
Oops – you first must agree to fill out the questionnaire! Thank you

**Policy label text:**
We need your consent to use your anonymous data and answers. THANK YOU!

**WAVE 3 Summer Semester 2024
Introduction Text:**
Hello! We're glad you're taking part in our survey! We're conducting a study on smoking and vaping. You can help us by completing this short questionnaire. There is no ‘right’ or ‘wrong’ answer – just answer the questions as best you can based on your opinion or knowledge. By providing your matriculation number and name, we can issue you with a TP certificate (0.25). You can participate if you are older than 17 years!
**IMPORTANT: You will receive a TP certificate for 0.25 TP if you enter your name and matriculation number at the end by your free choice. However, we will ask for your age, social media use and nicotine consumption – i.e. smoking or vaping.** **The results are intended for use in scientific publications to clarify fundamental questions of communication**. So you are doing something for science! The following scientists are asking: Prof. Regina Hanke (Design Professor at Macromedia - University of Applied Science Berlin), Dr. Andreas Hoheisel (Specialist in Pneumology, Pneumological Practice Leipzig / University Hospital Freiburg (Brsg.), Kwan-Young Yang (Statistician, Bad Homburg vor der Höhe) PD Dr. Thomas Köhnlein (Specialist in Pneumology, Pneumological Practice Teuchern). Thank you for completing the questionnaire – it should take about 10 minutes. Prof. Regina Hanke

**Privacy Policy Text:**
Background: This is a survey as part of an academic study. The questionnaire is answered anonymously –if you do not provide your Matrikel Number and Name on your own account. We do not collect any data that would allow you to be identified beyond this – no email, no names, no addresses. Duration of storage: In accordance with good scientific practice, the collected data will be stored for up to 10 years on the servers of Macromedia – University of Applied Science. The server is located in Germany. Use of the collected data: All collected data will be used for the following purposes within the framework of the scientific study: Project-related work, Scientific publications, Publications or exhibitions.

**Legal basis:** GDPR Art. 13 By participating in the questionnaire, you consent to the processing of your data. Responsible: Prof. Regina Hanke Design & Design management r.hanke@macromedia.de

**Privacy Policy Error Text:**
Oops – you first must agree that you are ready to complete the questionnaire! Thank you

**Privacy Policy label text:**
We need your consent to use your anonymous data and answers. THANK YOU!
