# Supplementary material for: Influence of social media channels on tobacco product consumption behaviors in people aged 18–30 years
Source: Front Public Health. 2026 Feb 23;14:1718804. doi: 10.3389/fpubh.2026.1718804 (PMC12968281; doi:10.3389/fpubh.2026.1718804)
Supplement: Supplementary file 3 [file Table_3.docx]

**Supplementary Table 1.** Descriptive Statistics for Platform Use and Content Consumption by Smoking Status

|  |  | N | Mean | SD | SE | 95% Confidence Interval for Mean | | Minimum | Maximum |
| --- | --- | --- | --- | --- | --- | --- | --- | --- | --- |
|  |  |  |  |  |  | Lower Bound | Upper Bound |  |  |
| Tik Tok | Non-smoker | 195 | 2.1385 | 1.2420 | 0.0889 | 1.9630 | 2.3139 | 1 | 5 |
|  | Smoker | 68 | 2.2059 | 1.2878 | 0.1562 | 1.8942 | 2.5176 | 1 | 5 |
|  | Vaper | 37 | 2.6486 | 1.3987 | 0.2300 | 2.1823 | 3.1150 | 1 | 5 |
|  | Total | 300 | 2.2167 | 1.2787 | 0.0738 | 2.0714 | 2.3620 | 1 | 5 |
| YouTube | Non-smoker | 195 | 2.3897 | 1.0061 | 0.0720 | 2.2476 | 2.5318 | 1 | 5 |
|  | Smoker | 68 | 2.4412 | 1.0979 | 0.1331 | 2.1754 | 2.7069 | 1 | 5 |
|  | Vaper | 37 | 2.0811 | 0.8938 | 0.1469 | 1.7831 | 2.3791 | 1 | 5 |
|  | Total | 300 | 2.3633 | 1.0172 | 0.0587 | 2.2478 | 2.4789 | 1 | 5 |
| Twitter | Non-smoker | 195 | 1.1641 | 0.4583 | 0.0328 | 1.0994 | 1.2288 | 1 | 4 |
|  | Smoker | 68 | 1.1471 | 0.3964 | 0.0481 | 1.0511 | 1.2430 | 1 | 3 |
|  | Vaper | 37 | 1.1622 | 0.5534 | 0.0910 | 0.9776 | 1.3467 | 1 | 4 |
|  | Total | 300 | 1.1600 | 0.4565 | 0.0264 | 1.1081 | 1.2119 | 1 | 4 |
| Instagram | Non-smoker | 195 | 2.5333 | 0.8691 | 0.0622 | 2.4106 | 2.6561 | 1 | 5 |
|  | Smoker | 68 | 2.6765 | 0.8884 | 0.1077 | 2.4614 | 2.8915 | 1 | 4 |
|  | Vaper | 37 | 2.8919 | 0.7740 | 0.1272 | 2.6338 | 3.1500 | 1 | 5 |
|  | Total | 300 | 2.6100 | 0.8682 | 0.0501 | 2.5114 | 2.7086 | 1 | 5 |
| Facebook | Non-smoker | 195 | 1.0923 | 0.3393 | 0.0243 | 1.0444 | 1.1402 | 1 | 3 |
|  | Smoker | 68 | 1.0735 | 0.2629 | 0.0319 | 1.0099 | 1.1372 | 1 | 2 |
|  | Vaper | 37 | 1.0811 | 0.2767 | 0.0455 | 0.9888 | 1.1733 | 1 | 2 |
|  | Total | 300 | 1.0867 | 0.3154 | 0.0182 | 1.0508 | 1.1225 | 1 | 3 |
| Knowledge | Non-smoker | 195 | 2.8821 | 0.6971 | 0.0499 | 2.7836 | 2.9805 | 1 | 4 |
|  | Smoker | 68 | 2.9853 | 0.7226 | 0.0876 | 2.8104 | 3.1602 | 1 | 4 |
|  | Vaper | 37 | 2.7838 | 0.5838 | 0.0960 | 2.5891 | 2.9784 | 2 | 4 |
|  | Total | 300 | 2.8933 | 0.6906 | 0.0399 | 2.8149 | 2.9718 | 1 | 4 |
| Gaming | Non-smoker | 195 | 1.7385 | 1.0737 | 0.0769 | 1.5868 | 1.8901 | 1 | 4 |
|  | Smoker | 68 | 1.7353 | 0.9867 | 0.1197 | 1.4965 | 1.9741 | 1 | 4 |
|  | Vaper | 37 | 2.6486 | 0.7534 | 0.1239 | 2.3975 | 2.8998 | 1 | 4 |
|  | Total | 300 | 1.8500 | 1.0605 | 0.0612 | 1.7295 | 1.9705 | 1 | 4 |
| News | Non-smoker | 195 | 2.6154 | 0.7935 | 0.0568 | 2.5033 | 2.7275 | 1 | 4 |
|  | Smoker | 68 | 2.7059 | 0.8297 | 0.1006 | 2.5051 | 2.9067 | 1 | 4 |
|  | Vaper | 37 | 1.6757 | 1.0289 | 0.1691 | 1.3326 | 2.0187 | 1 | 4 |
|  | Total | 300 | 2.5200 | 0.8901 | 0.0514 | 2.4189 | 2.6211 | 1 | 4 |
| Entertainment | Non-smoker | 195 | 3.3436 | 0.6421 | 0.0460 | 3.2529 | 3.4343 | 1 | 4 |
|  | Smoker | 68 | 3.3088 | 0.7776 | 0.0943 | 3.1206 | 3.4970 | 1 | 4 |
|  | Vaper | 37 | 3.4595 | 0.5052 | 0.0831 | 3.2910 | 3.6279 | 3 | 4 |
|  | Total | 300 | 3.3500 | 0.6600 | 0.0381 | 3.2750 | 3.4250 | 1 | 4 |
| Fashion & Beauty | Non-smoker | 195 | 2.4154 | 1.0388 | 0.0744 | 2.2687 | 2.5621 | 1 | 4 |
|  | Smoker | 68 | 2.2059 | 1.1138 | 0.1351 | 1.9363 | 2.4755 | 1 | 4 |
|  | Vaper | 37 | 2.7838 | 0.9468 | 0.1557 | 2.4681 | 3.0995 | 1 | 4 |
|  | Total | 300 | 2.4133 | 1.0549 | 0.0609 | 2.2935 | 2.5332 | 1 | 4 |

**Supplementary Table 2**. Post-Hoc Group Differences in Social Media Platform Use and Content Preferences by Smoking Status

|  |  | (I) Grouping | (J) Grouping | Difference  (I-J) | SE | p-value | 95% CI | |
| --- | --- | --- | --- | --- | --- | --- | --- | --- |
|  |  |  |  |  |  |  | Lower Bound | Upper Bound |
| Tik Tok | Tukey HSD | Non-smoker | Smoker | -0.0674 | 0.1792 | 0.9250 | -0.4895 | 0.3547 |
|  |  |  | Vaper | -0.5102 | 0.2282 | 0.0669 | -1.0476 | 0.0272 |
|  |  | Vaper | Smoker | 0.4428 | 0.2599 | 0.2056 | -0.1695 | 1.0550 |
| YouTube | Games-Howell | Non-smoker | Smoker | -0.0514 | 0.1514 | 0.9384 | -0.4112 | 0.3083 |
|  |  |  | Vaper | 0.3087 | 0.1636 | 0.1523 | -0.0856 | 0.7029 |
|  |  | Vaper | Smoker | -0.3601 | 0.1983 | 0.1703 | -0.8328 | 0.1126 |
| Twitter^1^ | Tukey HSD | Non-smoker | Smoker | 0.0170 | 0.0645 | 0.9623 | -0.1349 | 0.1690 |
|  |  |  | Vaper | 0.0019 | 0.0821 | 0.9997 | -0.1915 | 0.1954 |
|  |  | Vaper | Smoker | 0.0151 | 0.0936 | 0.9857 | -0.2053 | 0.2355 |
| Instagram | Games-Howell | Non-smoker | Smoker | -0.1431 | 0.1244 | 0.4853 | -0.4386 | 0.1523 |
|  |  |  | Vaper | -.3586* | 0.1417 | 0.0374 | -0.6998 | -0.0173 |
|  |  | Vaper | Smoker | 0.2154 | 0.1667 | 0.4037 | -0.1825 | 0.6133 |
| Facebook^1^ | Tukey HSD | Non-smoker | Smoker | 0.0188 | 0.0446 | 0.9068 | -0.0862 | 0.1237 |
|  |  |  | Vaper | 0.0112 | 0.0567 | 0.9786 | -0.1224 | 0.1449 |
|  |  | Vaper | Smoker | 0.0076 | 0.0646 | 0.9925 | -0.1447 | 0.1598 |
| Knowledge | Tukey HSD | Non-smoker | Smoker | -0.1032 | 0.0972 | 0.5385 | -0.3323 | 0.1258 |
|  |  |  | Vaper | 0.0983 | 0.1238 | 0.7071 | -0.1933 | 0.3899 |
|  |  | Vaper | Smoker | -0.2015 | 0.1410 | 0.3274 | -0.5337 | 0.1307 |
| Gaming | Games-Howell | Non-smoker | Smoker | 0.0032 | 0.1422 | 0.9997 | -0.3342 | 0.3405 |
|  |  |  | Vaper | -.9102* | 0.1458 | 0.0000 | -1.2596 | -0.5608 |
|  |  | Vaper | Smoker | .9134* | 0.1722 | 0.0000 | 0.5031 | 1.3236 |
| News | Games-Howell | Non-smoker | Smoker | -0.0905 | 0.1155 | 0.7141 | -0.3649 | 0.1839 |
|  |  |  | Vaper | .9397* | 0.1784 | 0.0000 | 0.5071 | 1.3723 |
|  |  | Vaper | Smoker | -1.0302* | 0.1968 | 0.0000 | -1.5028 | -0.5576 |
| Entertainment | Games-Howell | Non-smoker | Smoker | 0.0348 | 0.1049 | 0.9413 | -0.2148 | 0.2843 |
|  |  |  | Vaper | -0.1159 | 0.0949 | 0.4458 | -0.3440 | 0.1122 |
|  |  | Vaper | Smoker | 0.1506 | 0.1257 | 0.4567 | -0.1483 | 0.4496 |
| Fashion & Beauty | Tukey HSD | Non-smoker | Smoker | 0.2095 | 0.1473 | 0.3305 | -0.1374 | 0.5564 |
|  |  |  | Vaper | -0.3684 | 0.1875 | 0.1228 | -0.8101 | 0.0733 |
|  |  | Vaper | Smoker | .5779* | 0.2136 | 0.0197 | 0.0747 | 1.0811 |

1. For Twitter and Facebook, normality assumption violated.

**Supplementary Table 3**. An association exists between nicotine consumption behavior and social media channels (multinomial logistic regression results)

|  |  | **Category vs Reference** | | |
| --- | --- | --- | --- | --- |
|  |  | Smoker vs Non-smoker | Vaper vs Non-smoker | Smoker vs Vaper |
| **Predictor** | Estimate |  |  |  |
| TikTok | OR | 1.1267 | 1.0210 | 1.1036 |
|  | CI 95% | (0.8842 - 1.4355) | (0.7032 - 1.4821) | (0.7309 - 1.6662) |
|  | p-value | 0.3345 | 0.9132 | 0.6392 |
| YouTube | OR | 1.0328 | 0.5821 | 1.7743 |
|  | CI 95% | (0.7525 - 1.4172) | (0.3415 - 0.9920) | (0.9940 - 3.1668) |
|  | p-value | 0.8417 | 0.0466* | 0.0524 |
| X [Twitter] | OR | 0.8813 | 1.2414 | 0.7099 |
|  | CI 95% | (0.4516 - 1.7199) | (0.4837 - 3.1858) | (0.2444 - 2.0619) |
|  | p-value | 0.7112 | 0.6529 | 0.5288 |
| Instagram | OR | 1.2968 | 1.9052 | 0.6807 |
|  | CI 95% | (0.9244 - 1.8191) | (1.1119 - 3.2644) | (0.3779 - 1.2259) |
|  | p-value | 0.1323 | 0.019* | 0.2000 |
| Facebook | OR | 0.7964 | 1.0642 | 0.7484 |
|  | CI 95% | (0.3052 - 2.0776) | (0.2734 - 4.1416) | (0.1550 - 3.6115) |
|  | p-value | 0.6417 | 0.9285 | 0.7182 |
| Knowledge & Science | OR | 1.2253 | 1.3010 | 0.9418 |
|  | CI 95% | (0.7806 - 1.9232) | (0.6361 - 2.6608) | (0.4292 - 2.0660) |
|  | p-value | 0.3771 | 0.4710 | 0.8810 |
| Gaming | OR | 0.8545 | 2.7562 | 0.3100 |
|  | CI 95% | (0.5985 - 1.2199) | (1.7025 - 4.4619) | (0.1786 - 0.5380) |
|  | p-value | 0.3867 | < 0.0001* | < 0.0001* |
| News | OR | 1.0581 | 0.2836 | 3.7310 |
|  | CI 95% | (0.7216 - 1.5514) | (0.1591 - 0.5053) | (1.9628 - 7.0917) |
|  | p-value | 0.7724 | < 0.0001* | < 0.0001* |
| Entertainment | OR | 1.0080 | 1.3839 | 0.7284 |
|  | CI 95% | (0.6335 - 1.6036) | (0.6029 - 3.1761) | (0.2977 - 1.7818) |
|  | p-value | 0.9732 | 0.4434 | 0.4874 |
| Fashion & Beauty | OR | 0.7564 | 1.6758 | 0.4513 |
|  | CI 95% | (0.5242 - 1.0912) | (0.9298 - 3.0203) | (0.2353 - 0.8655) |
|  | p-value | 0.1355 | 0.0858 | 0.0166* |
| Gender (Male) | OR | 1.4133 | 1.3912 | 1.0159 |
|  | CI 95% | (0.6221 - 3.2102) | (0.3181 - 6.0841) | (0.2104 - 4.9039) |
|  | p-value | 0.4086 | 0.6610 | 0.9844 |

N = 300, Non-smokers 65%, Smokers 22.7%, Vapers 12.3%), * indicates significance at the 5% level.

Multinomial Logistic Regression Model Fit

| χ² | 86.248, df = 22, p <0.000 |
| --- | --- |
| Nagelkerke R² | 0.3023 |
| McFadden | 0.164 |
